# Chemical Composition, Cytotoxic, Apoptotic and Antioxidant Activities of Main Commercial Essential Oils in Palestine: A Comparative Study

**DOI:** 10.3390/medicines3040027

**Published:** 2016-10-25

**Authors:** Mohammad A. Al-Tamimi, Bob Rastall, Ibrahim M. Abu-Reidah

**Affiliations:** 1 Deptartment of Nutrition and Food Technology, An-Najah National University, PO Box 7, Nablus 415, Palestine; 2 Department of Food and Nutritional Sciences, The University of Reading, Whiteknights, Reading RG6 6AP, UK; r.a.rastall@reading.ac.uk; 3 Deptartment of Chemistry, An-Najah National University, PO Box 7, Nablus 415, Palestine; iabureidah@gmail.com or iabureidah@najah.edu

**Keywords:** essential oils (EOs), cytotoxicity, apoptosis, antioxidants, cell lines, anti-cancer activity

## Abstract

**Background:** Essential oils (EOs) are complex mixtures of several components gifted with a wide array of biological activities. The present research was designed to evaluate whether commercial essential oils could be effective by examining their in vitro antioxidant, cytotoxic, and apoptotic properties of nine commercially available EOs in Palestine, namely, African rue, basil, chamomile, fennel, fenugreek, ginger, spearmint, sage, and thyme, and to assure their effective use. **Methods:** The cytotoxic activity was determined using HT29-19(A) non-muco secreting and HT29-muco secreting (MS) cell lines. MTT, and trypan blue tests, and DPPH radical scavenging have also been assayed on the studied EOs. **Results:** In this work chamomile oil showed the lowest IC_50_ at the content of 60 µL/mL, while all other EOs reached such a decrease when 70–80 µL/mL was used on HT-29 (MS) cell lines. In HT-29 19(A) cells, 50% of viability was obtained when 80 µL/mL of ginger and African rue was used, while all other EOs needed more than 80 µL/mL to reach such a decline in viability. Otherwise, an MTT assay on HT-29 (MS) displayed ginger EO with the lowest IC_50_, followed by African rue and sage, with 40, 48 and 53 µL/mL, respectively. Otherwise, for the rest of the EOs, the IC_50_ was obtained by assaying around 80 µL/mL. Ginger showed the lowest IC_50_ with 60 µL/mL and thyme was the highest with 77 µL/mL when HT-29 19(A) cells were used. **Conclusion:** The most active EOs were found to be ginger, chamomile oil, and African rue. In general, the results demonstrate that most commercial EOs tested in this work possess low, or no biological activities; this may be due to processing, storage conditions, and handling or other reasons, which may cause losses in the biological and pharmacological properties that endemically exist in the Eos; hence, more investigation is still required on commercial EOs before they are recommended to the public.

## 1. Introduction

Aromatic plants (APs) have been used since antiquity as a potential source of drug discovery and development of disease chemoprevention, in folk medicine, and as preservatives in foods [1]. The best known aromatic plants: viz, chamomile, fennel, ginger, thyme, basil, and sage, originate from the Mediterranean area. The Middle East in general and Palestine in particular, are areas with many endemic plants whose compounds could be used in medicine [2,3]. Many APs are used for different industrial purposes such as food, drugs, and perfumery manufacturing.

APs contain many biologically active compounds, mainly phenolics and EOs, which have been found to possess antioxidant, antiparasitic, antimicrobial, anti-carcinogenic, and anti-inflammatory properties, among others [4]. Essential oils (EOs) are also used for the management of chronic diseases like cardiovascular, diabetes, Alzheimer’s, cancer, and others [5].

EOs are natural, concentrated, volatile aromatic compounds isolated from plants which have long been used in medicine, pharmaceutical, perfumery, cosmetic, and in many food applications. Initially, EOs have been utilized in medicine, but in the last decades their use as fragrance and essence ingredients has improved to become the major employment. Up to now, about 3000 EOs are well-known, of which, around 10% is engaged commercially in the flavor and aroma markets [6].

It is well-known that EOs are found in lots of APs, which possess a wide array of biological and pharmacological activities, which are associated with traditional and complementary medicine [4].

EOs are highly complex mixtures involving several tens to hundreds of different types of individual volatile compounds such as terpenoid, oxygenated terpenes, sesquiterpenes, and hydrocarbons, which are responsible for their characteristic aroma. EOs are hydrophobic liquids with a particular odor and taste aroma which are often poorly soluble in water. EOs have been widely used for their virucidal, bactericidal, fungicidal, anticancer, antioxidant, antidiabetic activities [7,8]. They are usually prepared by fragrance extraction techniques such as distillation (hydro or steam distillation), cold pressing, extraction (maceration), or by using of supercritical carbon dioxide extraction [9,10]. The biological activity of EOs is strictly linked to their chemical composition.

Table 1 presents a list of common EOs commercially available in Palestine and their sources.

Commercial EOs are of great interest for consumers with people being prescribed them as traditional remedies. Doses of 5 to 20 mL of EOs can be prescribed to people to relieve pains, headaches, joint arthritis and problems in the digestive system (personal observation).

Indeed, there is a relationship between the production of reactive oxygen species (ROS) to the origin of oxidation and inflammation that can lead to cancer [11]. Oxidative stress is a major contributor to the pathogenesis of a number of chronic diseases, that is why antioxidant behavior is one of the most commonly determined biological activities in extracts of plants such as EOs [12]. It has been reported that more than half of most cancer cases and their consequent deaths worldwide are potentially preventable [13]. Therefore, modification in dietary habits by increasing consumption of functional foods rich in antioxidants such as EOs is greatly supported [14].

The anticancer activity of EOs has been described in more than five hundred scientific reports; those first published dated to the 1960s [15].

So far, the effects of EOs have been investigated on glioblastoma, melanoma, leukemia and oral cancers, as well as on bone, breast, cervix, colon, lung, prostate, and uterus cancers [16]. Among the anticancer medications, 70% of drugs approved between 1940 and 2002 are either natural products or developed based on knowledge gained from natural products [17]. Most researchers who experimented on the anti-proliferation properties of natural compounds have examined the apoptosis process of certain cancer cells [18].

In spite of all the information available on several EOs, the investigation dealing with this kind of commercial product has been inadequate. Moreover, to the best of our knowledge, there are no reports available about in vitro cytotoxicity of these studied commercial EOs against HT29-19A non-muco secreting and HT29-muco secreting cell line types. Therefore, the aim of the present work has been to study and compare the antioxidant, cytotoxic, and apoptotic activities of nine commercially available EOs together with their in vitro anticancer activity assayed by methyl thiazol tetrazolium (MTT) and trypan blue tests, in order to evaluate their significance for traditional use.

## 2. Materials and Methods

### 2.1. Essential Oils

The following commercial EOs were purchased from a local market in the city of Tulkarm, Palestine in May 2015, which was the end of the season for most aromatic plants grown locally, without knowing the conditions of production of these EOs. They are: African rue, basil, chamomile, fennel, fenugreek, ginger, mint, sage, and thyme, which are widely used in Palestine. All oils were micro-filtered using 0.2 µM disc (Sartorius Stedim Biotech, Goettingen, Germany) in order to eliminate any impurity present in the EO, then EOs were kept at 4 °C in dark containers till they were used for the experiment.

### 2.2. Gas Chromatography-Mass Spectrometry (GC-MS)

The analysis of the commercial EOs was performed on a GC-MS HP model 5975B inert MSD (Agilent Technologies, J&W Scientific Products, Palo Alto, CA, USA), equipped with an Agilent Technologies capillary DB-5MS column (30 m length; 0.25 mm i.d.; 0.25 mm film thickness), and coupled to a mass selective detector (MSD5975B, ionization voltage 70 eV; all Agilent, Santa Clara, CA, USA). The carrier gas (He) was used at a 1 mL min^−1^ flow rate. The oven temperature program was as follows: 1 min at 100 °C ramped from 100 to 260 °C at 4 °C min^−1^ and 10 min at 260 °C. The component concentration was obtained by semi-quantification by peak area integration from GC peaks.

### 2.3. Cell Culture Maintenance and Preparation

Mucus-secreting HT29-MS and non-mucus-secreting HT29-A (19) cells were obtained from the cells and culture collection at the University of Reading, UK. All cells were cultured in Dulbecco’s modified Eagle medium (DMEM)-high glucose, contains sodium pyruvate (Life Technologies, Paisley, UK), supplemented with 10% defibrinated fetal bovine serum, 5% GlutaMAX™ (Life Technologies, Paisley, UK) and 1% antibacterial/antimycotic solution (Sigma-Aldrich, Pool, UK). All cells were grown in T-75 cm^2^ flask and incubated at 37 °C with 5% CO_2_ and 95% relative humidity. Media were changed every other day.

After reaching 70%–80% confluence, cells were split as follows; media were aspirated and cells were washed twice with 5 mL pre-warmed Phosphate buffered saline (Sigma-Aldrich, Pool, UK), then 5 mL trypsin-EDTA (0.5 g/L, Sigma-Aldrich, Pool, UK) was added and cells were re-incubated for a further 10 min. Trypsin was deactivated by adding 5 mL fresh medium and the suspension was centrifuged at 1800 rpm for 5 min. Supernatant was aspirated, then pelleted cells were reconstituted with 1 mL fresh medium. Cell count was conducted using a hemocytometer and microscopy and 105 cells/mL were recultured in a sterile flask. Flasks were monitored daily and checked microscopically for any contamination.

For the experiment, cells were cultured as above for 21 days and 15 days for HT29-(MS) and HT29-19 (A), respectively, so they reach maturation then they were used for viability, cytotoxicity and apoptosis tests.

### 2.4. Trypan Blue Exclusion Assay

To determine the effect of the EOs on the viability of cells, approximately, 105 mature cells/mL were transferred in a 12-well tissue culture plate and left for 48 h to establish adherence to plate before different concentrations of EOs were mixed with DMEM media and 500 µL/mL of such mixture were pipetted into each well. Some wells with media containing no EOs were used as control. After 24 h the media were aspirated and cells were trypsinized, collected and resuspended in an equivolume of 0.4% Trypan blue (Sigma-Aldrich, Pool, UK). This experiment was done in triplicates and repeated three times. The percentages of viable cells were counted using an inverted microscope and the percent of viability was determined in comparison with the control. Photos for cells under the microscope at different time intervals were taken using a Nikon™ Coolpix, 5400 digital camera (Nikon Inc., Melville, NY, USA).

### 2.5. MTT Cytotoxicity Assay

The in vitro cytotoxic activity of the EOs on HT29-19 (A) and HT29-(MS) was determined using the MTT (3-[4,5-dimethylthiazol-2-yl]-2,5-diphenyl tetrazolium bromide) assay as follows. Briefly, 200 µL/mL of medium containing cells at a density of 2 × 105 mature cells/mL were seeded in each well of a flat-bottom 96-well plate. Cells were permitted to adhere to the plate for 48 h. Then media were replaced with 180 µL/mL of various concentrations of the EOs (0%–100% of original EOs mixed with DMEM media) and incubated for 24 h. After that, MTT solution (0.5 mg/mL, Sigma-Aldrich) 20 µL/mL was added. Plates were incubated at 37 °C for another 4 h after which cultures were removed from incubator and the resulting formazan crystals were dissolve by adding an amount of MTT solubilizing solution (10% Triton X-100 with 0.1 N HCl in anhydrous isopropanol) equal to the original culture medium volume. All tests and analyses were run in triplicate. Pipetting up and down was required to completely dissolve the MTT formazan crystals. DMSO was used as the positive control and wells were left with no cells for the negative control. The absorbance of each well was determined by a spectrophotometer at dual wavelengths of 570 and 690 nm for the background on a multi-well plate reader with software (Tecan Group Ltd., Mannedorf, Switzerland). The viability percentage was calculated by the following formula: the concentration providing 50% inhibition (IC_50_) was calculated from a graph plotting inhibition percentage against different EOs concentration.

Each experimental condition was analyzed in triplicate, with three experiments for each EO. Growth inhibition was calculated as follows:

% Viability = (OD_sample_ − OD_blank_/OD_control_ − OD_blank_) × 100

### 2.6. DPPH Radical Scavenging Assay

DPPH (2,2-Diphenyl-1-picrylhydrazyl, Sigma-Aldrich, Pool, UK) radical scavenging activity was measured as described by Molyneux [21] with some modifications. Briefly, 0.5 mL of EO (8 mg/mL in methanol) was added to 1 mL of DPPH solution (20 mg/mL in methanol) freshly prepared. After shaking, the mixture was incubated for 15 min in darkness at room temperature and then absorbance was measured at 517 nm against a control (mixture without EO). Quercetin (Sigma-Aldrich, Pool, UK) was used as positive control. The inhibition percentage of free DPPH radicals (I%) was calculated following the formula:

Percentage of radical scavenging = (Abs_control_ − (Abs_sample_/Abs_control_)) × 100

where Abs_control_ is the absorbance of the control reaction (blank with methanol and DPPH) and Abs_sample_ is the absorbance of the sample reaction (essential oil diluted in methanol and DPPH). The sample concentration (in 1 mL reaction mixture) providing 50% inhibition was estimated by plotting the percentages of inhibition against essential oil concentrations (Table 2). All determinations were performed in triplicate.

Five hundred µL of EO (8 mg/mL in methanol) was added to 1 mL of DPPH solution (20 mg/mL in methanol) freshly prepared. After shaking, the mixture was incubated for 15 min in darkness at room temperature and then absorbance was measured at 517 nm against a control. Quercetin was used for comparison.

### 2.7. Apoptosis Assay

Depending on cytotoxicity results, ginger oil was selected for examining apoptosis property which was measured using caspase-3 activity kit (Abcam, Cambridge, UK) according to the instructions of the manufacturer. Briefly, apoptosis was induced in cells by adding ginger oil, and the cells were incubated for 2 h. In addition, a control culture without induction was concurrently incubated. Cells were pelleted and counted almost 1 × 10^6^ cells. Cells were also re-suspended in 50 μL of chilled cell lysis buffer and incubated on ice for 10 min. Finally, they were centrifuged for 1 min in a micro-centrifuge (10,000× *g*).

## 3. Results

### 3.1. EOs Composition

For the used commercial oils, the supplier provided no data about their contents or chemical analysis, which is presumed to be the company’s copyright. However, a simple chemical analysis was performed in order to have a gross estimate of the components of the employed essential oils as % composition (Table 3).

### 3.2. Viability Test by Trypan Blue

**Figure 1:**
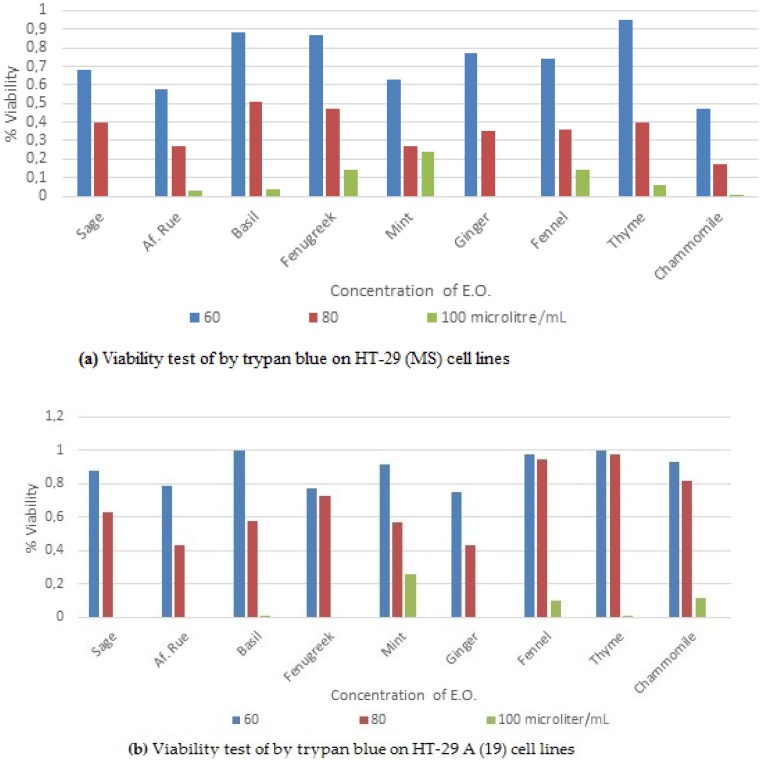
Viability percentages of: (**a**) HT29-19(A) non-muco secreting and (**b**) HT29-muco secreting (MS) cell lines treated for 48 h with EOs.

Viability test of both cell lines by trypan blue is shown in Figure 1a,b. HT-29 (MS) cell line viability decreased by 50% when 60 µL/mL of chamomile were used while all other EOs reached such a decrease when 70–80 µL/mL were used. In HT-29 19(A), 50% of viability was obtained when using 80 µL/mL of ginger and African rue, while all other EOs needed more than 80 µL/mL to reach such a decline in viability. However, apart from mint, all EOs produced 0% to 10% viability when concentrations were increased to 100 µL/mL.

**Figure 2:**
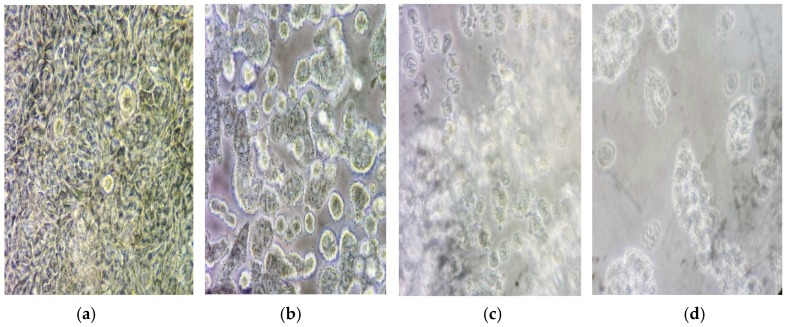
Effect of ginger oil on the HT-29 (MS) cell line as observed under the microscope. × 400 (**a**), represents cells at time 0 min; (**b**–**d**) cell at 30 min intervals after ginger oil addition.

Photos of ginger oil effect on cells at different time intervals are shown in Figure 2a–c.

### 3.3. MTT Assay

**Figure 3:**
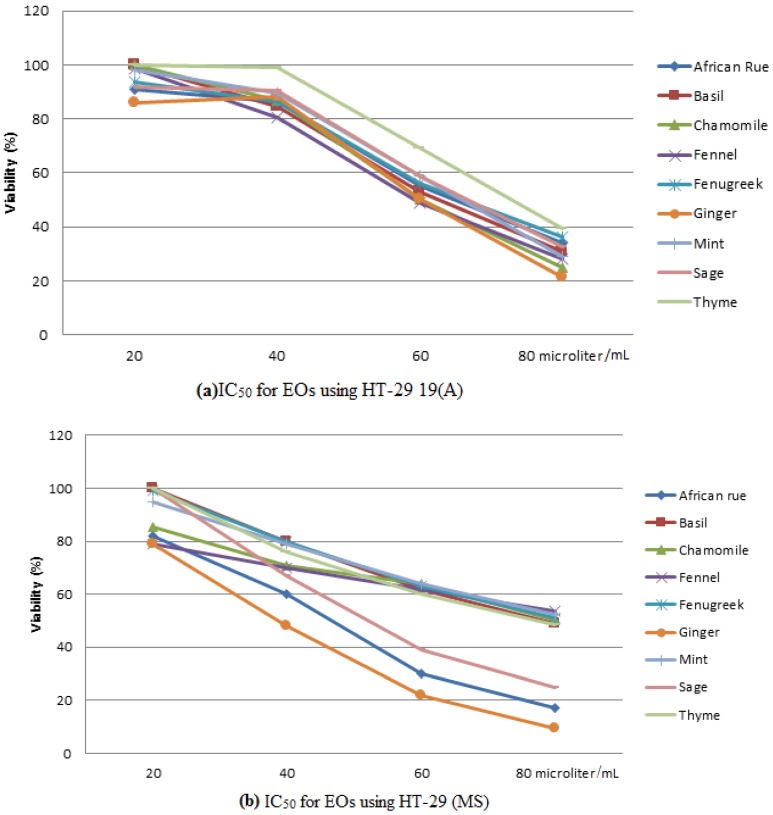
MTT assay using cell lines treated with EOs for 48 h: (**a**) IC_50_ on HT-29 19(A); (**b**) IC_50_ on HT-29 (MS).

The MTT cytotoxicity test for both cell lines is shown in Figure 3a,b. IC_50_ for EOs using HT-29 (MS) has revealed that ginger was the lowest in concentration to achieve IC_50_ followed by African rue and sage, with 40, 48 and 53 µL/mL respectively. Whereas, the rest of EOs showed IC_50_ at contents around 80 µL/mL. In the HT-29 19(A) cells, the same trend was obtained, in which ginger got the lowest concentration (60 µL/mL) then thyme was the highest with 77 µL/mL.

### 3.4. DPPH Radical Scavenging

The reduction ability of DPPH radicals’ formation was determined by the decrease in the absorbance at 517 nm induced by antioxidants. DPPH is a stable free radical and accepts an electron (hydrogen radical) to become a stable diamagnetic molecule. A DPPH assay revealed that all commercial EOs assayed in this study have got a very weak or no scavenging capacity compared with the control. These results may be a result of the low quality control of the production or the handling of these EOs products.

Scavenging activity was equivalent to ≤20 µg/mL quercetin, which was the lowest concentration used in the experiment.

### 3.5. Apoptosis

Ginger oil failed to show an ability to induce apoptosis within the time frame of this experiment. On the other side, caspase-3 activity has not been detected. The same was found for the rest of the EOs. This may be justified by the change in quality of the EOs composition due to many factors including the oxidation, adulteration, or aging [20,21]. As for the S-1 sample, the total amount was determined.

## 4. Discussion

As it can be noticed from the trypan blue experiment results, most of the EOs have been shown to have a similar trend to inhibit 50% growth (IC_50_) at the content of 80 µL/mL. Indeed, chamomile was very effective when used on HT-29 (MS), whereas, ginger and African rue showed superior effect in comparison with other EOs on HT-29 19(A) cells.

From the latest studies, ginger constituents were reported to have a vital effect in the control of tumor development through up-regulation of the tumor suppressor gene, induction of apoptosis and inactivation of VEGF pathways. For instance, 6-gingerol was found to have a role in the suppression of the hyper-proliferation, transformation, and inflammatory routes that take part in numerous steps of carcinogenesis, angiogenesis and metastasis; in addition, it acts in the initiation of apoptosis in the prostate cancer cell line via inhibition of cell invasion reduction of matrix metalloproteinase-9 expression. Also, 6-gingerol stimulates apoptosis through up-regulation of NAG-1 and G1 cell cycle arrest through down-regulation of cyclin D1 [22]. Besides, other abundant terpenoids also have been found to be present in the ginger like, neral, geranial, zingerberene, camphene, and other oxygenated monoterpenes [23] which may exert a synergistic anticancer activity.

Otherwise, *Peganum harmala* is traditionally used to treat many diseases including cancer. Recent studies show that the alkaloids of *Peganum harmala* are cytotoxic to several tumor cell lines in vitro and have an antitumor effect in a tumor model in vivo. Harmine, a major identified indole alkaloid in the African rue and vasicinone, were the most potent components in inhibiting cell growth and as an antiproliferating agent [24,25]. The active principle at a dose of 50 mg/kg given orally to mice for 40 days was found to have significant anti-tumoral activity [26].

The only difference between both cell lines was the ability of cells to secrete mucus. The negative effect of chamomile, in the case of HT-29 19 (A) cells, is not clear. Mucus in the gut plays a major role in protecting the gut linen from foreign (bio)chemicals. In vitro, a lack of secreting mucus may induce other protective mechanisms that enabled HT-29 19(A) to withstand the impact of higher concentration of EOs. However, as soon as this mechanism was damaged, the viability dramatically declined. On the other side, protection, by mucus, from the effect of the EOs in HT-29 (MS) has gradually decreased as the concentrations of the EOs were increasing.

Scavenging ability of antioxidants decreases by several factors, such as direct light exposure, storage temperature and processing (time needed in open air and temperature) [20]. Commercial EOs are treated and stored in a way in which they can lose their antioxidant and other biological properties. Indeed, there are inevitable factors due to oxidation in the extraction process and in the storage bulk scale of essential oil [20]. Moreover, in Palestine, some EOs are displayed on the shelves in transparent containers, and the temperature may exceed 35 °C in summer time. However, the compositional change in the EOs may be unavoidable even at 5 °C. Such bad preservation conditions may prevent the products from retaining their ability as free radical scavengers.

In fact, EOs which possess high levels of unsaturation might be generally unstable due to many factors such as heat, light, hydration and oxidation.

## 5. Conclusions

From the EOs tested in this study, we noticed that some had potential activities against the cancer cell tested. Interestingly, in this work, chamomile oil showed the lowest IC_50_ at a content of 60 µL/mL in HT-29 (MS) cell lines. In HT-29 19(A) cells, 50% of the viability was obtained when 80 µL/mL of ginger and African rue EOs were used. An MTT assay on HT-29 (MS) cells showed that ginger IC_50_ was the lowest, followed by African rue and sage, with 40, 48 and 53 µL/mL, respectively. On the other hand, ginger had the lowest IC_50_ with 60 µL/mL whilst thyme was the highest with 77 µL/mL in HT-29 19(A) cells. However, the used commercial EOs did not show any biological activities in the antioxidant and caspase-3 assays.

Factors such as processing, handling, and storage conditions may be responsible for the faintness in the biological and pharmacological properties of the commercial EOs, which originally possess significant biological activities, suggesting that more investigation on commercial Eos is required before recommending their use to the public.

## Figures and Tables

**Table 1:** A list of the main popular commercial EOs in Palestine.

Essential Oil	Plant Family	Traditional Use and Activity	Reference
African rue (*Peganum harmala* L.)	Nitrariaceae (Zygophyllaceae)	Coughs, hypertension, diabetes, asthma, jaundice, lumbago, and many other human ailments, skin and subcutaneous tumors, skin diseases, wounds and lice.	[10,19]
*Basil* (*Ocimum basilicum* L.)	Lamiaceae	Antifungal, physicochemical and insect-repelling activity, antiseptic (postpartum infections) depression, migraine, stomach and intestinal ache.	[11]
Chamomile (*Matricaria chamomilla* L.)	Asteraceae	Abscesses, allergies, arthritis, boils, colic, cuts, cystitis, dermatitis, dysmenorrhea, earache, flatulence, hair, headache, inflamed skin, insect bites, insomnia, nausea, neuralgia, PMS, rheumatism, sores, sprains, strains, stress, wounds.	[12]
Fennel (*Foeniculum vulgare* Mill.)	Apiaceae (Umbelliferae)	Fennel essential oil is used as flavoring agents in food products also used as a constituent in cosmetic and pharmaceutical products. Herbal drugs and essential oil of fennel have antispasmodic, diuretic, anti-inflammatory, analgesic and antioxidant effects are active for dyspeptic complaints, flatulence and bloating. The volatile oil showed antimicrobial and hepatoprotective activity.	[13]
Fenugreek (*Trigonellafoenum-graecum* L.)	Fabaceae	Diabetes, sexual weakness, stomach and intestinal pain. The oil in the seeds is used as a skin softener and emollient. Fenugreek essential oil is rich in terpenenes.	[14,15]
*Ginger* (*Zingiber officinale* R.)	Zingiberaceae	wide application in flavor and perfumery industries, anti-emetic effect or control of nausea and vomiting, prevention of coronary artery disease, healing and prevention of both arthritic conditions and stomach ulcers.	[16]
Spearmint (*Mentha spicata* L.)	Lamiaceae	Food, cosmetic, confectionary, chewing gum, toothpaste and pharmaceutical industries. strong insecticidal and mutagenic activity.	[17]
Sage (*Salvia fruticosa* Mill.)	Lamiaceae	Antibacterial, cytostatic, antiviral and antioxidant activities. Moreover, they are frequently used in traditional medicine to treat diarrhea, eye diseases, gonorrhea; they possess antiseptic and antispasmodic activities. Also, the essential oils of Salvia species are used as cosmetics and as flavoring agents in perfumery.	[18]
Thyme (*Thymus vulgaris* L.)	Lamiaceae	Natural antimutagen	[20]

**Table 2:** DPPH Scavenging activity of the tested EOs.

Commercial EOs	African Rue	Basil	Chamomile	Fennel	Fenugreek	Ginger	Mint	Sage	Thyme
Quercetin equivalent (µg/mL)	20	20	20	20	<20	<20	<20	<20	<20

**Table 3:** Main chemical components of the investigated EOs using GC-MS.

Components of EOs	A. Rue (%)	Basil (%)	Chamomile (%)	Fennel (%)	Fenugreek (%)	Ginger (%)	Mint (%)	Sage (%)	Thyme (%)
(E)-Anethol	13								
Eugenol	20								
Cicloysosativene	5								
3-Decanone	1								
α-Isomethyl-(E)-ionol	7								
Carvone	2						80		
Carvacrol	6								8
Dihydrocarvenyl acetate							1		
Caryophyllene	3	1			15		1		
Neral (cis-citral)					17	9			
Methyl chavicol		76							
Limonene	1						9		15
Linalool		15							
Thymol	7								25
ρ-Cymene									14
α-Pinene				1	2		0.5		12
β-Pinene					15				
Anethole				75					
Fenchone				13					
*Cis*-thujone								30	
Camphor	3				17			22	
1,8-Cineole								8	
α-Selinene					4.5				
Geranial					5	10			
2,5-Dimethylpyrazine					7				
α-Bisabolol oxide A			24						
Chamazulene			10						
α-Bisabolone oxide A			19						
α-Bisabolol oxide B			30						
Spathulenol			4						
α-Zingiberene						17.4			
Camphene						8			
α-Farnesene						6			
β-Sesquiphellandrene						6.6			
Total identified chemicals	66%	92%	87%	89%	82.5%	57%	91.5%	60%	74%
